# Autoreactivity and Exceptional CDR Plasticity (but Not Unusual Polyspecificity) Hinder Elicitation of the Anti-HIV Antibody 4E10

**DOI:** 10.1371/journal.ppat.1003639

**Published:** 2013-09-26

**Authors:** Kathryn A. K. Finton, Kevin Larimore, H. Benjamin Larman, Della Friend, Colin Correnti, Peter B. Rupert, Stephen J. Elledge, Philip D. Greenberg, Roland K. Strong

**Affiliations:** 1 Division of Basic Sciences, Fred Hutchinson Cancer Research Center, Seattle, Washington, United States of America; 2 Program in Immunology, Cancer Research Division, Fred Hutchinson Cancer Research Center, Seattle, Washington, United States of America; 3 Department of Genetics, Harvard University Medical School, and Division of Genetics, Howard Hughes Medical Institute, Brigham and Women's Hospital, Boston, Massachusetts, United States of America; 4 Department of Immunology, University of Washington, Seattle, Washington, United States of America; 5 Department of Medicine, University of Washington, Seattle, Washington, United States of America; University of Zurich, Switzerland

## Abstract

The broadly-neutralizing anti-HIV antibody 4E10 recognizes an epitope in the membrane-proximal external region of the HIV envelope protein gp41. Previous attempts to elicit 4E10 by vaccination with envelope-derived or reverse-engineered immunogens have failed. It was presumed that the ontogeny of 4E10-equivalent responses was blocked by inherent autoreactivity and exceptional polyreactivity. We generated 4E10 heavy-chain knock-in mice, which displayed significant B cell dysregulation, consistent with recognition of autoantigen/s by 4E10 and the presumption that tolerance mechanisms may hinder the elicitation of 4E10 or 4E10-equivalent responses. Previously proposed candidate 4E10 autoantigens include the mitochondrial lipid cardiolipin and a nuclear splicing factor, 3B3. However, using carefully-controlled assays, 4E10 bound only weakly to cardiolipin-containing liposomes, but also bound negatively-charged, non-cardiolipin-containing liposomes comparably poorly. 4E10/liposome binding was predominantly mediated by electrostatic interactions rather than presumed hydrophobic interactions. The crystal structure of 4E10 free of bound ligands showed a dramatic restructuring of the combining site, occluding the HIV epitope binding site and revealing profound flexibility, but creating an electropositive pocket consistent with non-specific binding of phospholipid headgroups. These results strongly suggested that antigens other than cardiolipin mediate 4E10 autoreactivity. Using a synthetic peptide library spanning the human proteome, we determined that 4E10 displays limited and focused, but unexceptional, polyspecificity. We also identified a novel autoepitope shared by three ER-resident inositol trisphosphate receptors, validated through binding studies and immunohistochemistry. Tissue staining with 4E10 demonstrated reactivity consistent with the type 1 inositol trisphosphate receptor as the most likely candidate autoantigen, but is inconsistent with splicing factor 3B3. These results demonstrate that 4E10 recognition of liposomes competes with MPER recognition and that HIV antigen and autoepitope recognition may be distinct enough to permit eliciting 4E10-like antibodies, evading autoimmunity through directed engineering. However, 4E10 combining site flexibility, exceptional for a highly-matured antibody, may preclude eliciting 4E10 by conventional immunization strategies.

## Author Summary

4E10 is an example of an anti-HIV, broadly neutralizing antibody that is uncommon in infected patients and has not been successfully elicited by any vaccine approach attempted. 4E10 has been proposed to neutralize HIV through a mechanism that requires broad recognition of other antigens, including membrane phospholipids. Such a mechanism would also block the generation of 4E10 during B cell development, confounding vaccination strategies. Analysis of B cell development in 4E10 heavy-chain knock-in mice confirmed that 4E10 does recognize self-antigens. However, a previously proposed autoantigen candidate, the mitochondrial lipid cardiolipin, was not consistent with binding studies which showed that while 4E10 does bind liposomes containing cardiolipin, it does so only weakly and nonspecifically, also binding liposomes without cardiolipin. Using a synthetic human peptidome, 4E10 was shown to be polyreactive, binding peptides from various proteins, but only in a limited manner. Three of the top five hits are from types 1, 2 and 3 inositol trisphosphate receptors, with high scoring peptides sharing a conserved sequence motif. Validation of the top hits was performed by binding analyses and staining of tissue sections, which combined to identify the type 1 inositol trisphosphate receptor as the most likely 4E10 physiological autoantigen.

## Introduction

An effective prophylactic AIDS vaccine will need to generate anti-HIV neutralizing antibodies (Abs) that target the HIV envelope glycoprotein (Env) [1]–[3] and broadly neutralize as many HIV isolates as possible (bNAbs). The bNAb 4E10 [4]–[10] recognizes an epitope that is highly conserved across HIV-1, HIV-2, and SIV and displays one of the widest breadths of any anti-HIV bNAb, neutralizing 98% of HIV-1 strains [11], [12]. These properties have made 4E10 an attractive vaccine target, but previous attempts to elicit 4E10 or equivalent Abs through vaccination have failed.

The HIV envelope protein (Env) consists of gp120 surface subunits and gp41 membrane-anchoring subunits assembled as noncovalent trimers of gp120/gp41 heterodimers to form mature, functional ‘spikes’ on the virion surface. 4E10 recognizes a conserved linear epitope (consensus clade B sequence: ^671^ NWFDITNWLW ^680^; core epitope: NWF^D^/_N_IT), immediately adjacent to the viral membrane in the gp41 membrane proximal external region (MPER) [4], [10]. bNAbs targeting the MPER, such as 4E10 and a second bNAb, 2F5, are uncommon in infected individuals, and Env-derived immunogens do not efficiently elicit these Abs in vaccinees [13], [14]. 2F5 recognizes a linear epitope (consensus clade B sequence: ^662^ ELDKWA ^667^) neighboring the 4E10 epitope in the MPER [15]. It has been proposed that these Abs are inherently polyspecific and autoreactive as a direct consequence of their epitope specificity and neutralization mechanism, which has been posited to require interactions with lipids and viral membrane components in addition to and outside of their peptide epitopes [16]–[20]. Therefore, B cell tolerance mechanisms would limit the natural production of MPER-specific bNAbs, like 2F5 and 4E10, in people infected with HIV and hinder the elicitation of equivalent bNAbs by vaccination. Structural elements of 4E10 that have been proposed to mediate lipid or liposome recognition include the long, hydrophobic heavy chain complementarity determining region 3 (HCDR3) sequence and, in particular, the side-chain of residue W100H, which protrudes from the tip of HCDR3 at a position predicted to penetrate the viral membrane when bound to Env, based on the 4E10/epitope peptide complex crystal structure [10] and mutagenesis studies [21], [22].

Polyspecificity is the property of recognizing multiple distinct ligands (recognition degeneracy) combined with the specificity with which each of those ligands is recognized [23], [24]. Polyspecificity is particularly important for the function of adaptive immunoreceptors (T cell receptors and B cell receptors (BCRs)), which are functionally enhanced by broadly recognizing foreign antigens. Autoreactivity is the potential downside of unchecked polyspecificity, although autoreactivity does play a role in B cell activation and regulation (reviewed in [25]–[27]). On the order of 50% of newly-rearranged BCRs are estimated to be autoreactive and some degree of self-reactivity is necessary to pass through positive selection during B cell development; self-recognition by the pre-BCR induces the expansion of precursor cells and broadens antibody diversity. Autoreactivity can be defined functionally as dysregulation of B cell development (*e. g.* the developmental arrest, loss of immature B cells to central tolerance mechanisms and reduced numbers of residual splenic B cells with low surface IgM density observed in homozygous 2F5 V_H_DJ_H_ knock-in mice [28]) or *in vitro* with binding assays or immunofluorescence (IF) staining [29].

*In vivo* experiments demonstrating functional 4E10 autoreactivity had not been reported when we started these studies. 2F5 and 4E10 were originally concluded to be polyspecific and autoreactive on the basis of binding assays against 11 purified lipidic and nuclear autoantigens [16], [19]. 2F5 and 4E10 also both showed HEp-2 cell reactivity, exhibiting diffuse cytoplasmic and weaker nuclear staining patterns [19]. On the basis of these results, the 4E10 autoantigen was proposed to be the mitochondrial diphosphatidylglycerol lipid cardiolipin (CL) [30], [31], though 4E10 also showed comparable cross-reactivity against every lipid tested, including phosphatidylserine (PS), phosphatidylcholine (PC), phosphatidylethanolamine (PE) and sphingomyelin (SM). However, detection of clinically-relevant anti-CL autoantibodies (ACLA) by simple binding assays with isolated antigens, as has been previously reported [16], is associated with high false positive rates, representing challenges in the laboratory characterization of sera and Abs and the clinical evaluation of autoantibody induced morbidity [32]. More quantitative assays have been contradictory, with an estimated equilibrium dissociation constant (*K*_D_) in the low micromolar range in one study [18] but weaker, unquantifiable binding in others [33], [34]; 4E10 was reported to bind to model viral liposomes which did not contain CL with a sub-micromolar *K*_D_ [21], but showed no binding to ‘bald’, proteolytically-digested virus-like particles [35]. The disagreement of these results is due, in part, to employed methodologies, such as surface plasmon resonance (SPR), that can be difficult to reproduce and hard to evaluate in the absence of positive controls and when analyzing interactions with lipidic species [36].

Abs against phospholipids, including ACLA, are seen in primary antiphospholipid syndrome (APS) or in association with other autoimmune diseases, such systemic lupus erythematosus (SLE). ACLA in this context generally do not recognize CL in isolation, but as complexes with the phospholipid-binding serum protein β2 glycoprotein I (β2GPI) or other proteins with anti-coagulant activity. The presence of ACLA in these conditions can provoke serious complications, including arterial and venous thromboses and recurrent miscarriages. A second class of antiphospholipid antibodies can be generated after certain viral and bacterial infections, such as HIV, hepatitis B and C, syphilis and leprosy. ACLA are found in approximately 50% of people infected with HIV (*versus* ∼2% of uninfected controls) and are strongly linked with the level of viral replication, the level of B cell activation and the level of MPER-specific Abs [37], [38]; however, these Abs are usually not associated with APS manifestations and are not dependent on β2GPI for binding [39]. If 4E10 CL autoreactivity is sufficiently strong to trigger B cell tolerance mechanisms, it might be expected that 4E10 would contribute to APS; however, 4E10 showed only weak activity in APS clinical assays [33] and passive infusion studies [40], similar to that of antiphospholipid antibodies elicited during infections rather than that of ACLA.

While polyreactive across phospholipids, subsequent experiments with larger antigen arrays (a microarray of 106 connective tissue disease-related autoantigens plus controls [41] or the UNIchip AV-400 panel of 400 bacterially-expressed human proteins [33]) concluded that anti-HIV bNAbs were either not exceptionally polyreactive, including 2F5, 4E10 and the anti-CD4 binding site antibody b12, or displayed only limited polyreactivity, including 2F5 and 4E10 [42], [43]. The reactivity profile of 4E10 was, therefore, concluded to be fundamentally distinct from those of pathogenic autoantibodies that would trigger B cell tolerance mechanisms [41]. However, a recent analysis of 4E10 binding to a commercially-available array (Invitrogen ProtoArray 5) of 9,000 intact human proteins, expressed in baculovirus as GST-fusion proteins, concluded that 4E10 displayed “exceptional polyreactivity” on the basis of very broad, but low-avidity binding [20].

In this report, we found that knocking-in the 4E10 heavy chain into murine B cells results in comparable levels of dysregulation in the B cell compartment as had been observed in 2F5 heavy chain knock-in mice, demonstrating significant inherent autoreactivity, which was confirmed by clear patterns of specific autoreactivity by immunohistochemistry (IHC) on murine tissue sections. We determined that 4E10 does not stain mitochondria in HEp-2 cells *in vitro* or in tissue sections and that 4E10 bound only relatively weakly, but comparably, to liposomes containing CL or virus-like compositions lacking CL in well-controlled SPR binding assays using improved methodologies [36]. Therefore, 4E10 phospholipid binding is relatively weak, not specific for CL compared to other phospholipids, and distinct from properties of true ACLA responses. In order to isolate the 4E10 structural elements that might mediate general phospholipid binding activity, binding studies were performed with engineered HCDR3-grafted ubiquitin (Ubq) fusion constructs, with 4E10 point mutants, and at varying ionic strengths, which showed that the weak phospholipid binding activity displayed by 4E10 is driven by electrostatic interactions, not hydrophobic ones. The overall positive charge of the 4E10 Fv cassette, a property of many anti-HIV bNAbs [44], does not account for this behavior, which is likely alternately driven by an electropositive pocket present in the dramatically restructured combining site of ligand-free 4E10 but absent in epitope bound-state structures. However, 4E10, in either conformational state (free or complexed), lacks structural features required for truly specific recognition of CL. We therefore conclude that CL is unlikely to be the physiologically-relevant self-antigen mediating 4E10 autoreactivity and deletion during B cell development.

Because 4E10 was clearly autoreactive in 4E10 knock-in mice, we sought to identify candidates for *bona fide* 4E10 autoantigens using a synthetic library of 413,611 36-mer peptides spanning the human proteome combined with phage immunoprecipitation sequencing (PhIP-Seq) [45]. The results identify a short list of protein candidates, validated by *in vitro* binding studies and consistent with IHC results. The PhIP-Seq results were also useful for more finely defining 4E10 polyspecificity beyond previous studies, leading to the conclusion that 4E10 displays limited, highly focused polyspecificity, unexceptional for this class of molecules. Our findings should inform future attempts to generate 4E10-equivalent functionalities by vaccination and determine the precise molecular mechanism of 4E10 neutralization of HIV.

## Results

### Impaired B cell development and function in 4E10 heavy chain (4E10H) knock-in mice

**Figure 1:**
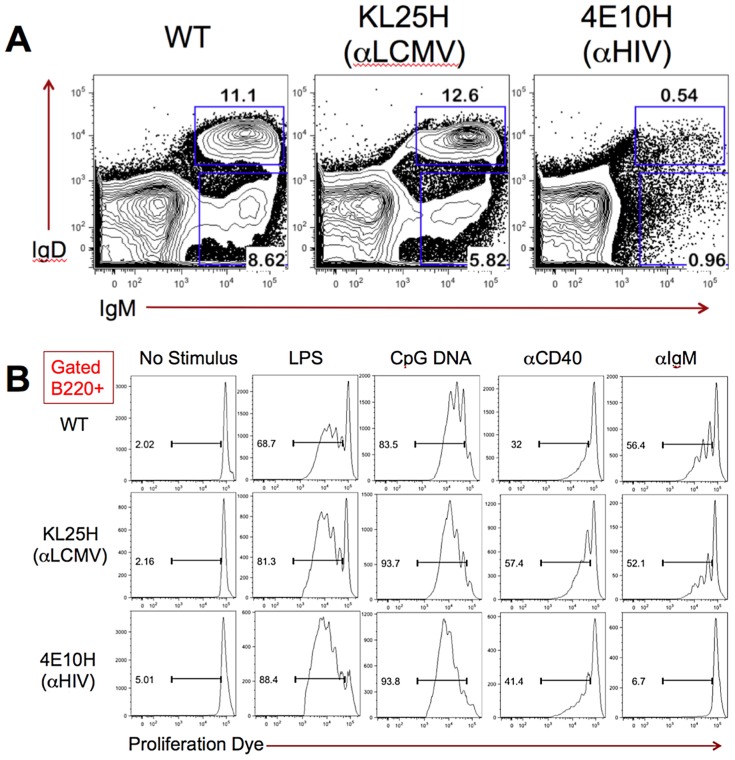
Analysis of 4E10H knock-in mice. (**A**) The percentages of IgM^+^IgD^−^ pre-B and IgM^+^IgD^+^ immature B cells within the B220^+^ cell population in the bone marrow of B6 WT, B6-KL25H and B6-4E10H mice are shown. Marrow cells were isolated and stained for cell surface markers as described in the methods section. FACS plots were previously gated B220^+^ and on live cell size based on forward and side scatter. Numbers represent the percentages of B220^+^ bone marrow cells falling within each box gate. (**B**) The proliferation of B220^+^ splenocytes from B6 WT, B6-KL25H, and B6-4E10H mice in response to overnight culture in the presence of B cell stimuli are shown. Cells were loaded with cell proliferation dye before overnight incubation, and proliferation was assessed by FACS analysis after cell surface staining to identify B220^+^ B cells. Histograms were previously gated B220^+^ and on live cell size based on forward and side scatter. Numbers represent the percentage of B220^+^ cells falling within the bar gate on each plot, indicating dilution of the proliferation dye as a result of cell division.

In order to evaluate the potential self-reactivity of 4E10, the rearranged human IgH VDJ antigen-binding domain of the 4E10 gene was targeted to the mouse immunoglobulin heavy chain (IgH) locus in B6 embryonic stem cells. B cell development in the derived 4E10H^+/+^ knock-in mice was evaluated by flow cytometry and compared with both wild-type B6 and control KL25H^+/+^ knock-in mice (**Fig. 1A**). KL25H^+/+^ knock-in mice express from the mouse IgH locus the rearranged mouse IgH VDJ antigen-binding domain of the LCMV-neutralizing antibody [46]. Flow cytometric analysis of bone marrow-resident B220^+^ cells from 4E10H^+/+^ knock-in mice revealed a dramatic reduction of both B220^+^IgM^+^IgD^−^ pre-B cells and B220^+^IgM^+^IgD^+^ immature B cells when compared with B6 mice. In contrast, KL25H knock-in mice displayed normal populations of developing B cells. These results suggest that expression of the 4E10 Ig heavy chain led to deletion of the majority of developing B cells as a result of self-antigen recognition.

Although there was a profound defect in B cell development, low numbers of B220^+^ B cells could still be found in the spleens and lymph nodes of 4E10H knock-in mice, suggesting that some 4E10H-expressing B cells had escaped deletion during development. To determine whether putative autoreactivity had impaired the function of 4E10H-expressing B cells, we tested the ability of these cells to respond to mitogenic and antigen receptor stimuli in vitro. Splenocytes were purified from 4E10H^+/+^, KL25H^+/+^, and WT B6 mice, loaded with cell proliferation dye eFlour670, and cultured overnight in the presence of LPS, CpG DNA, an activating anti-CD40 antibody, anti-IgM F(ab′)_2_, or in culture media alone (no stimulus). The following day, B cell proliferation was assessed by flow cytometric measurement of dye dilution in B220^+^ cells (**Fig. 1B**). B220^+^ B cells from 4E10H, KL25H, and WT mice placed in culture media alone remained highly stained with proliferation dye, indicating they had not undergone cell division. 4E10H^+/+^, KL25H^+/+^, and WT B6 B cells diluted the dye after treatment with LPS, CpG DNA, or anti-CD40, indicating that these cells had undergone cell division. This suggested that 4E10H-expressing B cells were capable of responding to a variety of mitogenic signals and did not have a general cellular activation defect. However, while both KL25H^+/+^ and B6 B cells proliferated in response to IgM cross-linking, 4E10H^+/+^ B cells did not, suggesting that these cells were anergic to antigen receptor signaling, a phenotype consistent with self-antigen reactivity.

### 4E10 bound liposomes only weakly, but predominately through electrostatic interactions

Having confirmed that 4E10H was autoreactive in a murine background, we sought to characterize 4E10 interactions with CL in order to determine definitively whether the affinity and specificity for this phospholipid biochemically met criteria for autorecognition. We tested binding of 4E10 IgG to liposomes captured on SPR biosensors with three different compositions approximating (*i*) the external leaflet of cellular membranes (PC/cholesterol), (*ii*) membranes containing CL (PC/cholesterol/CL) or (*iii*) the HIV viral envelope (PC/SM/PE/PS/cholesterol [22]). We also incorporated improvements into these SPR protocols compared to previous analyses. Liposomes were prepared with biotinylated lipids (0.5% w/w) and captured on streptavidin-coated sensor chips (Biacore SA chips (GE)) [47] rather than adsorbing prepared liposomes to lipophilic groups covalently attached to carboxymethylated dextran-coated sensor chips (Biacore L1 chips (GE)). This approach reduces the need to block dextran lipid groups with either saturating amounts of liposomes or directly chip-coupled bovine serum albumin (BSA), improving signal-to-noise ratios, and has been determined to generate much more consistent and reliable results than L1 sensor chips [36] or ELISA-based methods [32], [48]. BSA was added to all analyte buffers throughout analysis to act as a blocking reagent, preventing nonspecific binding. Annexin V, which binds to both CL and PS [37], was used as a positive control in order to provide comparative responses. Double subtraction [49] was also used to correct sensorgrams, which were run in duplicate, where both reference cell and buffer-matched blank responses are considered.

**Figure 2:**
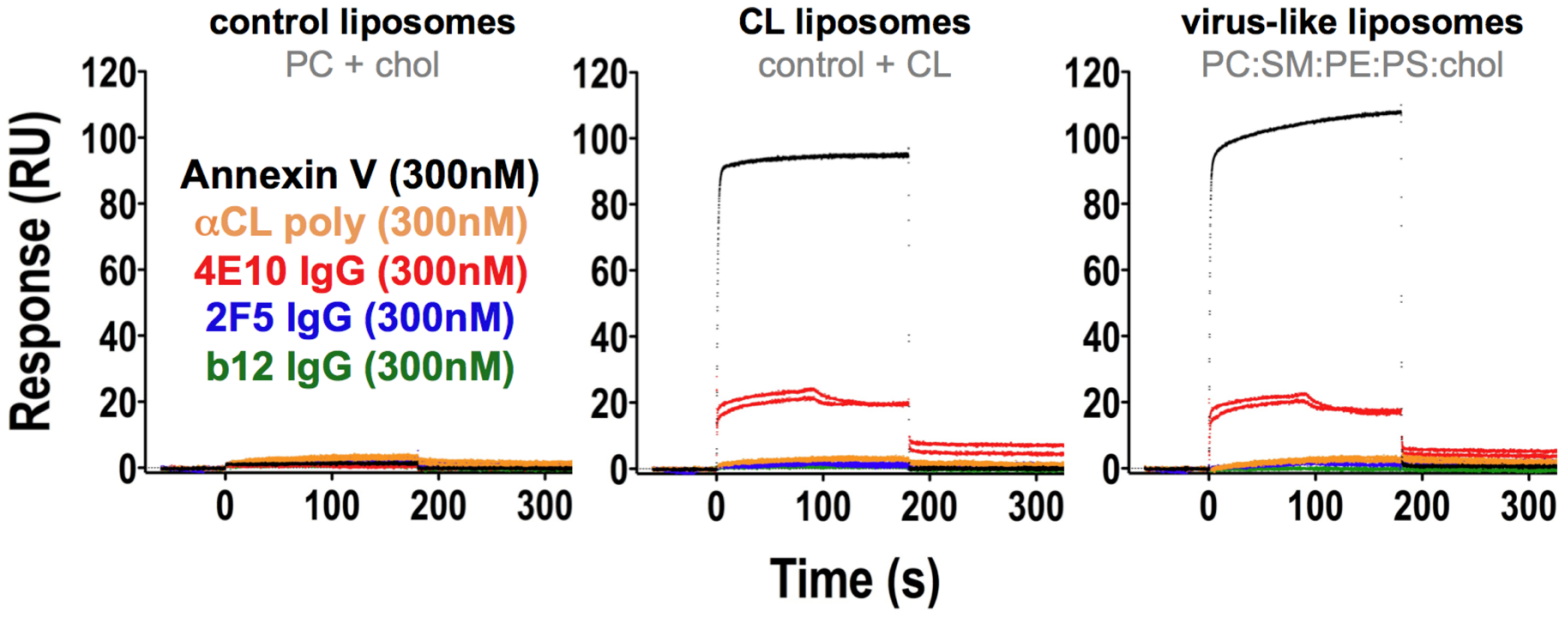
SPR analyses of 4E10/liposome interactions. Corrected SPR responses are shown (response units: RU; duplicate runs) of Annexin V (black), αCL polyclonal Ab (orange), 4E10 IgG (red), 2F5 IgG (blue), and b12 IgG (green) analytes binding to liposomes incorporating biotinylated lipids captured on streptavidin-coated biosensor chips. All analyte concentrations were 300 nM. The Annexin V analyte buffer included 2.5 mM CaCl_2_. Liposome compositions are indicated above each frame.

These combined improvements result in SPR sensorgrams that show cleanly negative results for the binding of Abs and Annexin V to control liposomes (PC/cholesterol) but clear, reproducible, positive results for Annexin V binding to liposomes incorporating CL or PS (**Fig. 2**). 2F5 and b12 monoclonal IgGs and a patient-derived ACLA polyclonal antiserum did not bind appreciably to liposomes of any composition tested. However, 4E10 IgG showed weak, but reproducible binding to CL liposomes containing PC, cholesterol and CL and virus-like liposomes containing PE, PC, PS, SM and cholesterol, but lacking CL. However, while detectable, the SPR binding responses, which reflect bivalent avidities, could not be quantified accurately, due to the weak responses and distinct, biphasic association profiles unfittable by any reasonable model. Nevertheless, 4E10 binding responses to CL and virus-like liposomes were qualitatively comparable, demonstrating a lack of specificity for CL over other negatively-charged phospholipids. 4E10 IgG, the bivalent form, was used in these experiments because univalent reagents, like scFvs [34], did not display detectable binding (*data not shown*), confirming the weakness of these interactions in absolute terms.

**Figure 3:**
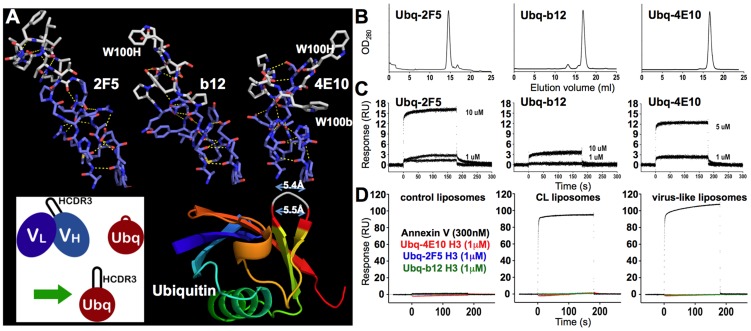
Design and characterization of HCDR3-grafted Ubq fusion proteins. (**A**) A ribbon representation of Ubq (1UBQ.pdb [106]) is shown at bottom right, colored prismatically from N- (blue) to C-terminus (red); the targeted loop for HCDR3 engraftment is highlighted in gray with red bounding residues. The structures of the three targeted HCDR3 segments in context of their parent Abs are shown above in a licorice-stick representation, colored by atom type (oxygen: red; nitrogen: blue; carbons of buried residues: marine blue; and carbons of exposed residues: gray). Residue exposure was determined with the program GetArea [107], with greater than 30% solvent accessibility considered “exposed”, based on the structural context in the corresponding crystal structures (2F5: 3IDG.pdb [108]; b12: 3RU8.pdb [104]; 4E10: 2FX7.pdb [9]). The three transferred sequences (2F5: AHRRGP**T**T**LFGVPIARG**PVNAMDVW; b12: ARVG**PYSWDDSP**QYNYYMDVW; 4E10: AREGTT**GWGWLGKP**IGAFAHW; exposed residues **bolded**), were characterized as “hydrophobic” with the Sigma-Aldrich PEPscreen Library Design Tool (Φ_2F5_ = 0.52; Φ_b12_ = 0.51; Φ_4E10_ = 0.56). Key residues and Cα-Cα distances (Å) are indicated. The inset shows a schematic representation of the design process. Molecular images were generated with MacPyMOL [109]. (**B**) SEC analyses of the solution properties of the purified recombinant HCDR3-grafted Ubq fusion proteins are shown, confirming monodispersivity. (**C**) Corrected SPR responses (duplicate runs) of HCDR3-grafted Ubq fusion proteins to HIV SF162 gp140 protein amine-coupled to biosensor chips; analyte concentrations are indicated. While detectable, these responses are consistent with *K*_D_ values >>10 µM and are thus too weak to quantify reliably. (**D**) SPR responses (duplicate runs) of Annexin V and HCDR3-grafted Ubq fusion protein analytes to liposomes incorporating biotinylated lipids captured on streptavidin-coated biosensor chips are shown, with liposome compositions indicated above each frame. HCDR3-grafted Ubq fusion protein responses are colored as indicated, but none showed detectable binding on any liposome composition.

It had been hypothesized that the HCDR3 of 4E10 makes direct hydrophobic contacts with the viral membrane, facilitating binding through aromatic and apolar amino acid side-chains, particularly W100H and W100bH [21]. In order to test whether or not 4E10 HCDR3 alone supports liposome binding, HCDR3-grafted Ubq fusions were designed (**Fig. 3A**). Ubq is a small, stable protein with an exposed loop (residues 46–47) bracketed by residues spaced by the same distance as the framework residues bracketing HCDR3 in Abs. The fusions were generated by replacing this two residue loop in Ubq with the HCDR3 sequences of 2F5, b12 or 4E10. The three Ubq fusion constructs, expressed in bacteria, were monodisperse in solution after purification, determined by size-exclusion chromatography (SEC; **Fig. 3B**), and showed detectable, but weak binding to chip-coupled HIV SF162 gp140 by SPR (**Fig. 3C**), confirming their utility for biochemical assays. However, all three HCDR3-grafted Ubq fusions failed to show any binding response by SPR to liposomes of any composition tested (**Fig. 3D**), even at relatively high analyte concentrations, showing that hydrophobic HCDR3 sequences alone, out of context of the rest of the folded Ab structure, are insufficient to confer even minimal binding to liposomes.

**Figure 4:**
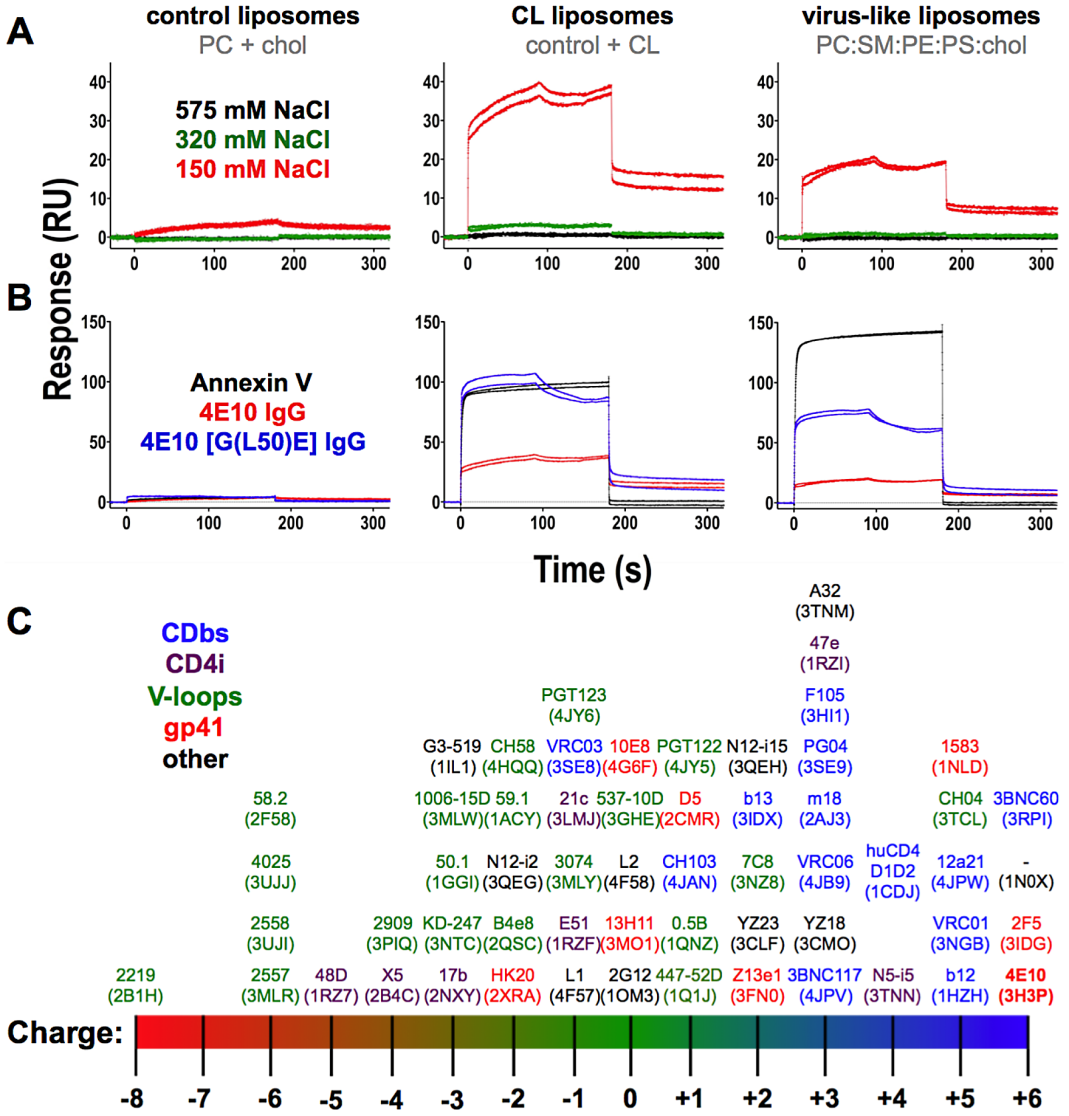
Analysis of the effects of salt concentration, mutation and overall cassette charge on 4E10/liposome interactions. Corrected SPR responses are shown for Annexin V or 4E10 IgG analytes (300 nM; duplicate runs) to liposomes incorporating biotinylated lipids captured on streptavidin-coated biosensor chips; liposome compositions are indicated above each frame. (**A**) SPR responses of wild-type 4E10 IgG analytes at different salt concentrations are plotted. (**B**) SPR responses of Annexin V (300 nM), wild-type 4E10 IgG (300 nM) or the 4E10 [G(L50)E] mutant IgG (400 nM) are plotted. A higher concentration of 4E10 mutant IgG was used with the expectation that binding might be significantly reduced. Since this did not occur, mutant IgG responses appear elevated due to the concentration differential. (**C**) The net charge at neutral pH of the Fv cassettes of the anti-HIV Abs with structures currently available through the PDB [103] was calculated with the structure-based algorithm PDB2PQ [51]–[54]. Two Abs, PG16 and NIH45-46, were excluded because their structures included modified amino acids that could not be accommodated by PDB2PQ. Fvs are plotted with their names, with assigned PDB accession codes in parentheses. Ab labels are colored by the locale of their epitopes on Env, as indicated; 4E10 is also bolded.

Biomolecular interactions are mediated by hydrophobic, electrostatic, van der Waals and hydrogen bonds. The widely-held presumption has been that 4E10 binds lipids, liposomes and membranes through predominately hydrophobic interactions mediated by HCDR3 and other combining site residues. In order to determine the overall character of 4E10/liposome binding, SPR analyses were conducted at a series of salt concentrations; increasing the salt concentration drives hydrophobic interactions but ablates electrostatic ones, which is the fundamental principle behind ion exchange and hydrophobic interaction chromatography. SPR analyses showed that 4E10 IgG interactions with both CL-containing and virus-like liposomes were abolished by increasing the salt concentration (**Fig. 4A**), demonstrating that 4E10 interacts with liposomes containing phospholipids with negatively-charged headgroups (*e. g.* CL) predominately through electrostatic interactions, not hydrophobic ones.

It had previously been suggested that 4E10 contains a phosphate-binding subsite [50] and that a structurally-reasonable phosphate binding site is present on the light chain side of the combining site, bracketed by the side-chains of K32L and K100eH [34]. In the latter study, a 4E10 [G(L50)E] IgG mutant was constructed that places a glutamic acid side-chain directly between the K32L and K100eH side-chains, designed to disrupt phosphate binding. In order to identify features on 4E10 that could mediate phospholipid interactions outside of hydrophobic combining site residues, the [G(L50)E] mutant was tested for liposome binding by SPR (**Fig. 4B**). However, 4E10 [G(L50)E] IgG retained binding to liposomes containing CL and virus-like liposomes, demonstrating that mutational ablation of the K32L/K100eH site does not ablate phospholipid binding. Outside of the K32L/K100eH site, inspection of available 4E10 structures, which were all determined as complexes with HIV epitope-related ligands, failed to reveal any obvious electropositive pocket, crevice or groove that might be reasonably inferred to confer CL binding, so no further targeted mutagenesis studies were performed. Structure-based calculations of the net charges at neutral pH of the Fv cassettes of all available anti-HIV antibody structures using PDB2PQ [51]–[54], showed that 4E10 and 2F5 have exceptional net positive charges (**Fig. 4C**). However, since this level of overall electropositivity of 2F5 is insufficient to confer liposome binding, it is also unlikely to account for 4E10 liposome binding.

### The crystal structure of unbound 4E10 revealed a completely restructured combining site

**Figure 5:**
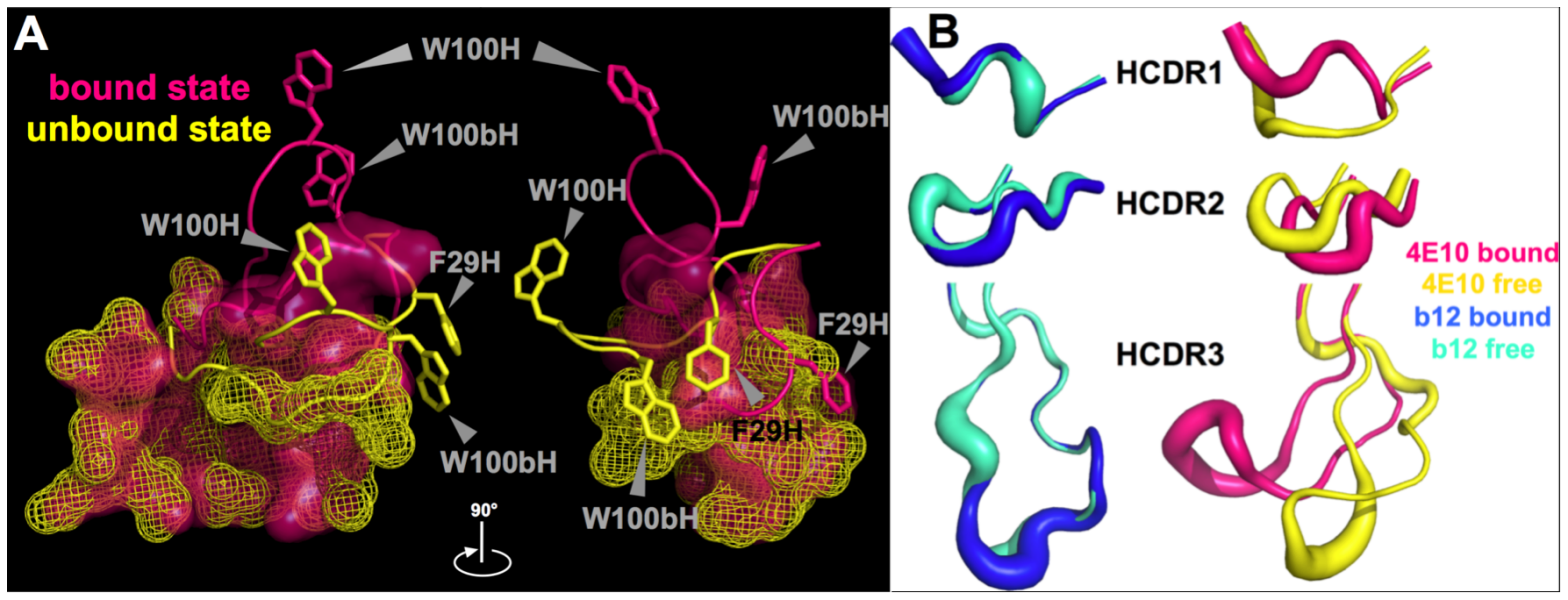
Structure of 4E10 free of bound antigen. (**A**) Superposition of the three HCDRs from bound and unbound b12 (3RU8.pdb [104], 1HZH.pdb [110] and 4E10 (2FX7.pdb [9], 4LLV.pdb) are shown in a B-factor putty representation (b12, blue: bound; cyan: unbound. 4E10, pink: bound, yellow: unbound). (**B**) Superposition of residues from the 4E10 epitope binding site and HCDR1 and 3 from bound (semi-transparent molecular surface in pink; 2FX7.pdb [9]) and unbound (mesh molecular surface in yellow; 4LLV.pdb) 4E10 Fv are shown; HCDR1 and 3 are shown in cartoon representations with side-chains of key residues shown in licorice-stick representations and labeled. The molecule is oriented so that the V_L_ domains are to the left and the V_H_ domains to the right. Molecular images were generated with MacPyMOL [109].

The species actually binding lipids in our studies was 4E10 free of bound ligand, where all previous 4E10 structures were determined as complexes with MPER epitope-related ligands. While highly affinity-matured Abs tend to have rigidified combining sites [55]–[67], we sought to crystallize free 4E10 to identify potential structural flexibility that might explain these discrepancies in the absence of alternative hypotheses. The 4E10 Fv in the unbound state was crystallized from Li_2_SO_4_ (d_min_ = 2.4 Å), preliminary phases were determined by molecular replacement, and the structure was rebuilt and refined with good agreement statistics and geometry (**Table 1**). The asymmetric unit contains four copies of 4E10, grouped as two pairs related by a single NCS two-fold axis, arranged in a pinwheel-like pattern with the HCDR3s forming the central spoke. V_L_ domains of Fvs from one member of each dyad-related pair are relatively poorly ordered, likely due to sparse crystal contacts. Overall, the Fv domains show a high degree of structural conservation to each other, with the main differences arising from alternate HCDR1 and HCDR3 conformations between dyad-related pairs. The HCDR3 conformation of one dyad-related pair is extended into a solvent channel and unconstrained by crystal packing, with large accompanying crystallographic B-factors, likely closely recapitulating the free, solution-state structure (**Fig. 5A**). The HCDR3 conformation from the second dyad-related pair is alternatively scrunched by extensive crystal packing and hydrophobic interactions across the plane bisecting the dyad axis, resulting in low B-factors stemming from these steric constraints. Comparison of the presumed solution-state, unbound 4E10 conformer, using the better-ordered dyad mate, with a reference epitope-peptide 4E10 Fab complex structure (2FX7.pdb [9]), showed that the largest structural differences are also between HCDR1 and 3, with movements of ∼12 Å between the Cα positions of W100H in HCDR3 and ∼6 Å between the Cα positions of F29H in HCDR1 (**Fig. 5A and B**; pair-wise superpositions resulted in a root mean square deviations (RMSD) on all common Cαs of 0.76 Å for the heavy chain, with HCDR1 and 3 deleted, and 0.32 Å for the light chain). The movements of HCDR1 and 3 reposition the side-chains of F29H and W100bH, which are both buried in the V_H_ domain in epitope complexes, to form hydrophobic interactions partially covering the side-chains of L53H, L54H and I56H in HCDR2. These three side-chains are exposed in the epitope complex structures, making contacts with a hydrophobic face of the MPER epitope. In the complex structures, HCDR3 forms the back wall of the epitope binding pocket. In the unbound structure, however, HCDR displacements remodel the 4E10 combining site, completely occluding epitope binding but creating a shallow concavity on the backside of HCDR3, a surface typically buried in Ab structures. One of the ordered sulfates modeled into the structure sits near the middle of this concavity, coordinated by the guanidinium group of R94H and main-chain and side-chain contacts from S28H.

**Table 1:** Crystallographic data collection and refinement statistics.

	Synchrotron	In-house
**Data collection** *		
Space group	*C*222_1_	*C*222_1_
Cell dimensions		
*a*, *b*, *c* (Å)	88.89, 161.6, 163.3	88.89, 161.6, 163.3
Resolution (Å)	46.1-2.39 (2.44-2.39)	50.0-2.35 (2.43-2.35)
*R*_merge_	0.072 (0.382)	0.066 (0.438)
*I*/σ*I*	28.45 (5.6)	22.97 (2.66)
Completeness (%)	100.0 (99.8)	99.9 (99.9)
Redundancy	8.3 (3.0)	4.1 (2.5)
**Refinement**		
No. reflections (unique)	46,684 (2,305)	49,263 (4,863)
*R*_work_/*R*_free_	0.216/0.245	–
No. non-hydrogen atoms		
Protein	6,452	–
Ligand/ion	81	–
Water	296	–
*B*-factors (Å^2^)		
Protein	42.48	–
Ligand/ion	40.99	–
Water	26.12	–
R.m.s. deviations		
Bond lengths (Å)	0.006	–
Bond angles (°)	0.899	–
Ramachandran plot statistics (MolProbity)		
Residues in most favored regions (%)	97.6%	–
Residues in disallowed regions (%)	0.0%	–
MolProbity score	1.37	–
Est. coordinate error (max. likelihood ESUc; Å)	0.161	–
Accession code	4LLV	

* Values in parentheses are for the highest-resolution shell.

### 4E10 did not stain HEp-2 cells by IF microscopy

**Figure 6:**
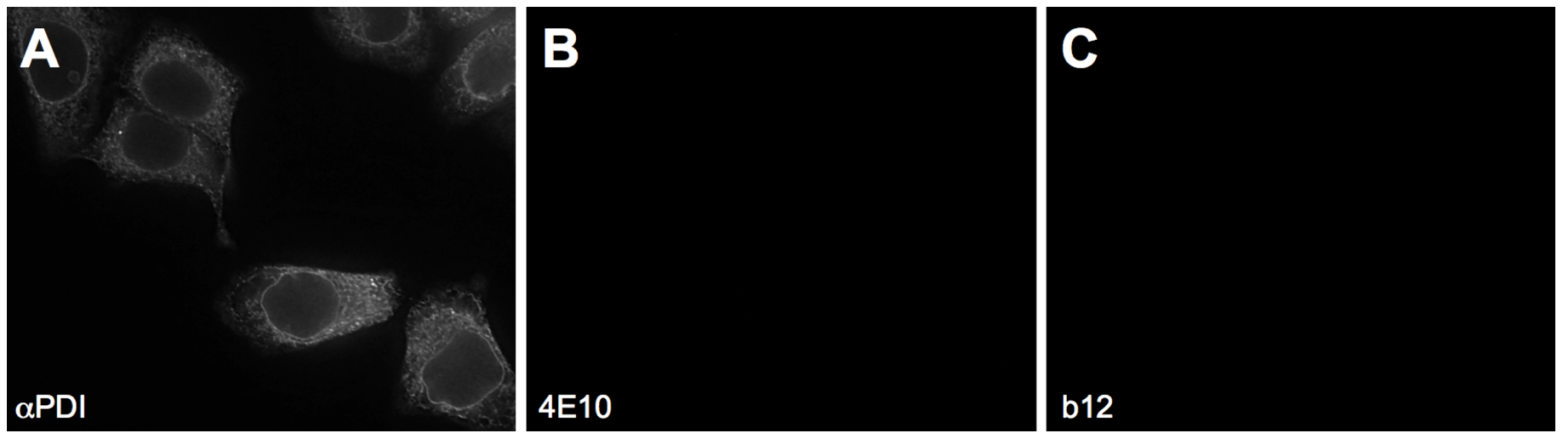
IF microscopy of HEp-2 cells. Fixed and permeabilized HEp-2 cells were stained with (**A**) αPDI IgG (an ER marker), (**B**) 4E10 IgG, or (**C**) b12 IgG, appropriate fluorescently-labeled secondary Abs, and visualized microscopically. Image parameters were carefully matched across experiments.

4E10 had been reported to show diffuse cytoplasmic and nuclear staining of HEp-2 cells [19], [20], suggesting the presence of a cross-reacting autoantigen. Higher resolution confocal microscopy of HEp-2 cells stained with 4E10 IgG was used to expand the prior result by narrowing the subcellular localization and begin the process of identifying the antigen/s. Fixed and permeabilized HEp-2 cells were incubated with 4E10 or, as comparative controls, b12 or an anti-protein disulfide isomerase (αPDI) IgG (**Fig. 6**). b12 has been reported to stain HEp-2 cells in a similar pattern, though with stronger nucleolar staining in the nucleus, but this Ab has not been reported to be autoreactive [16], and αPDI marks the endoplasmic reticulum (ER). If 4E10 can bind CL with a physiologically relevant affinity, 4E10 would be predicted to stain cells with a mitochondrial pattern. The αPDI IgG stains HEp-2 cells in the expected ER pattern (**Fig. 6A**), but 4E10 and b12 bind irreproducibly in replicate experiments and failed to show any specific staining (**Figs. 6B and C**), negating this approach.

### Analysis of 4E10 autoreactivity and polyspecificity using a synthetic human peptidome

**Figure 7:**
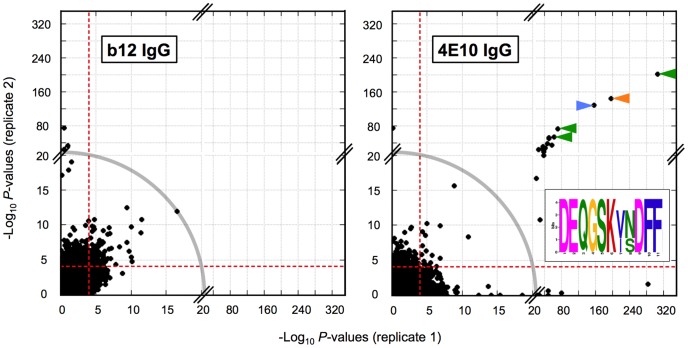
PhIP-Seq analyses of 4E10 and b12. PhIP-Seq results are plotted as −Log_10_ *P* values, one replicate on the abscissa, the other on the ordinate; note the discontinuity in axis scales. The top five scoring 4E10 peptides are highlighted with arrows; the three peptides derived from IP_3_R isoform sequences are highlighted with green arrows, with the consensus core sequence shown as a MEME logo [72], [111] in the inset. The orange arrow corresponds to a peptide derived from cytoplasmic dynein 1 and the blue arrow to a peptide derived from complement receptor type 1. The gray arc corresponds to the −Log_10_ *P* values observed for the highest-scoring b12 peptide, so defines a threshold for potential autoreactive binding events. Proximity to the diagonal indicates good replicate concordance; peptides with highly discordant replicate values, falling along the axes, outside the bounding dashed red lines were discarded from the analysis.

**Figure 8:**
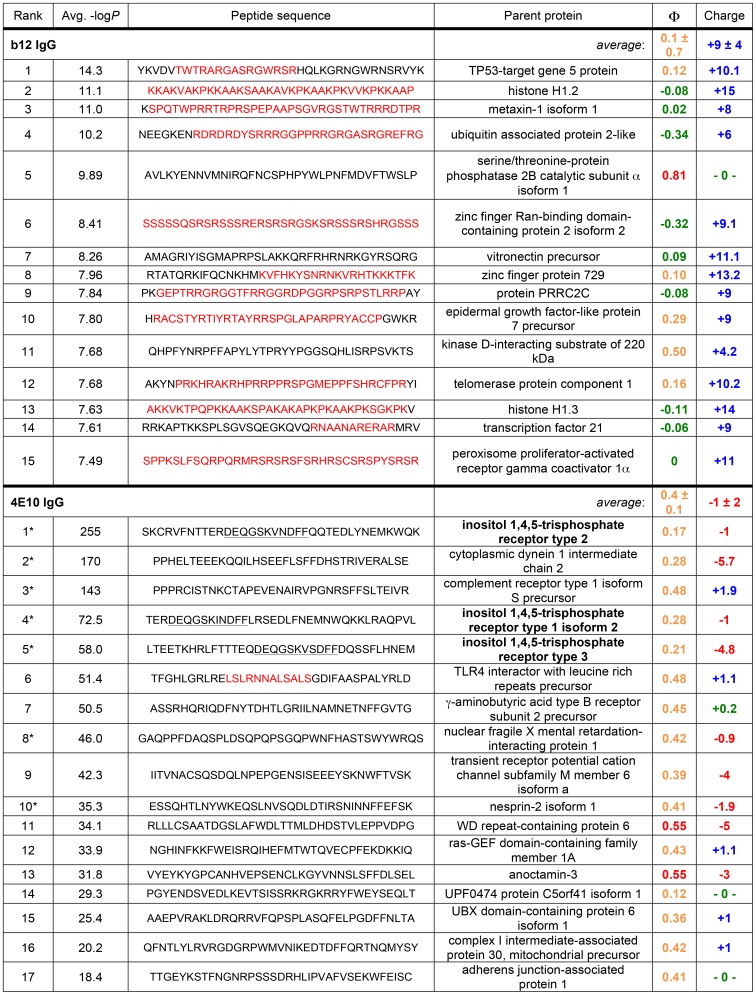
Top scoring 4E10 and b12 PhIP-Seq 36-mer peptides. The top fifteen scoring 36-mer peptides from the b12 IgG PhIP-Seq analysis (above) and the seventeen top scoring 36-mer peptides from the 4E10 IgG analysis exceeding the threshold set by the highest-scoring b12 peptide (below) are shown, compiled by their relative rank order, replicate-averaged PhIP-Seq score, sequence, identity of the parent protein, hydrophobicity (Φ) and predicted charge at neutral pH. Hydrophobicities were determined with the Sigma-Aldrich PEPscreen Library Design Tool (green: hydrophilic; orange: moderately hydrophobic; red: hydrophobic; no peptides were predicted to be “very hydrophobic”); overall charge was determined with the Innovagen Peptide Property Calculator (red: negative; green: neutral; blue: positive). Segments of low sequence complexity (defined by the program SEG [112]) are highlighted in red; the core motif in the IP_3_R isoform peptides (labeled in bold), identified by MEME [72], is underlined. Asterisks indicate peptide sequences selected for expression as fusion constructs for subsequent validation.

A phage-displayed library of 413,611 overlapping 36-mer peptides spanning the complete human proteome [45] was used to identify candidate autoantigens and assess the polyspecificity of 4E10; b12 was also analyzed for comparison (**Figs. 7** and **8**). In this library, neighboring peptides in contiguous open reading frames overlap by seven residues, the approximate size of a linear Ab epitope. Both Abs were analyzed in duplicate, with results plotted as replicate #1 *versus* replicate #2 −Log_10_ *P*-values [45]. Plotted in this way, highly discordant replicate pairs lie near the axes and were discarded from further consideration; 241 36-mers were also culled because of nonspecific binding. Based on sequence searches, no sequence motifs associated with the core epitope of 4E10 (NWF^D^/_N_IT) or alternate proposed HIV epitopes [68] are present in the human proteome, so positive binding results in these assays reflect truly polyspecific recognition of distinct epitopes. b12 showed results consistent with a non-autoreactive Ab of limited polyspecificity (**Fig. 7A**), with the highest scoring peptide having a replicate average −Log_10_ *P*-value equal to 14.3 and with approximately two dozen peptides rising above the main cluster (**Figs. 7A** and **8**). Detailed inspection of the 15 top-scoring b12 36-mer peptides (**Fig. 8**) showed a consistent pattern of highly positively-charged peptides (average predicted net charge at neutral pH of +9) with blocks of low sequence complexity in 12 of 15 peptides.

The 4E10 PhIP-Seq results were distinct from the b12 results (**Figs. 7B** and **8**). Using the highest scoring b12 peptide as a benchmark under the assumption that b12 is not autoreactive, 17 36-mer peptides showed significantly higher scores (replicate average −Log_10_ *P*-values of 18.4 to 255). The chemical character of these 17 4E10 peptides was distinct, as well: overall moderately hydrophobic or hydrophobic (though none were very hydrophobic), with a range of predicted net charges at neutral pH from −5.7 to +1.9 and greater sequence complexity. None of the 17 high-scoring peptides had sequences related to HIV, as expected, but we were able to identify a conserved motif. Three of the five top-scoring peptides, including the highest scoring peptide, are derived from an ER-resident inositol trisphosphate receptor (IP_3_R; NP_002214 [69], [70]) [71]. MEME motif analysis [72] revealed a highly conserved core motif (DEQGSK^V^/_I_^N^/_S_DFF) within these three peptides quite unrelated to the 4E10 MPER epitope (**Fig. 7B** inset). While no homologous crystal structure is available, Phyre^2^ threading [73] predicts that this sequence forms part of an extended helix in IP_3_R. The other two top-five scoring 36-mers were from cytoplasmic dynein 1 intermediate chain 2 (NP_001369 [69], [70]) and the complement receptor type 1 isoform S precursor (NP_000642 [69], [70]). None of the peptides from the top two scoring proteins from the previously reported ProtoArray 5 analysis [20] (retinoid X receptor β and interleukin-1 family member 6) or the proposed autoantigen, splicing factor 3B3 (SF3B3; ProtoArray 5 rank not reported), had average −Log_10_ *P*-values above 1.54 in the PhIP-Seq analysis (4E10 library average = 0.27 (σ = 0.82); b12 library average = 0.33 (σ = 0.45)). The close concordance in library scoring behavior also argues against the presence of very broad, low-avidity binding by 4E10 relative to b12.

### Validation of PhIP-Seq top-scoring peptides by SPR

**Figure 9:**
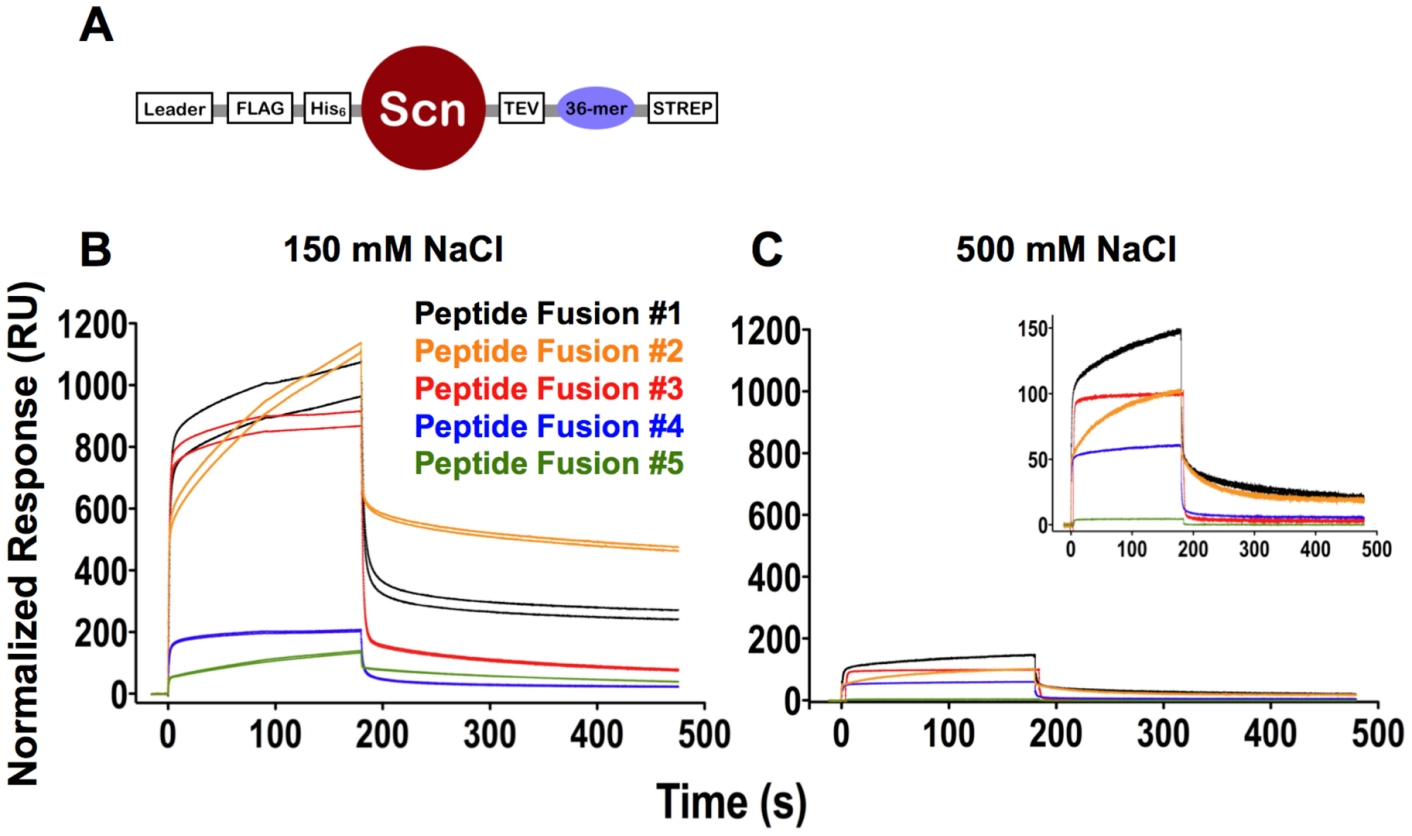
SPR analyses of Scn-peptide fusions. (**A**) The generalized siderocalin fusion construct used for PhIP-Seq peptide expression is shown. (**B**) Corrected and normalized SPR responses for 4E10 IgG binding to the top five peptides immobilized on a CM5 biosensor chip are plotted. (**C**) Corrected and normalized SPR responses of 4E10 IgG (300 nM; duplicate runs) binding to peptides (colored as in (**B**)) at 500 mM NaCl are plotted; inset shows magnified view. Normalized SPR responses are plotted in arbitrary units.

The five top scoring peptides identified by PhIP-Seq (**Fig. 8**) were further validated by testing for binding to 4E10 directly by SPR. Peptides were expressed in a eukaryotic secretion-pathway expression system as fusion constructs with the human protein siderocalin (Scn; **Fig. 9A**) [74]; expression in bacterial systems resulted in unacceptable levels of proteolytic degradation (*data not shown*). The constructs incorporated various purification and epitope-tags, including a C-terminal Strep-tag [75]. Western blots (*data not shown*) and SPR analyses with an anti-Strep-tag Ab confirmed the presence of full length peptides, with specific activities assayed by SPR of 12% to 34% as coupled, which allowed for normalizing results between SPR experiments (**Figs. 9B and C**). All five peptides showed definitively positive binding results (**Fig. 9B**), whereas no binding was seen to the siderocalin construct minus peptide, confirming specific interactions. Binding was reduced by 75% to 85%, but not ablated, in the presence of 500 mM NaCl (**Fig. 9C**), indicating that the interactions were not exclusively electrostatic in nature and drawing a distinction with 4E10 recognition of liposomes. The qualitative, normalized SPR responses monotonically decreased from peptide #1 to #5 to fairly weak values, so no further peptide fusions from hits farther down the PhIP-Seq list were evaluated.

### IHC showed specific patterns of 4E10 staining

**Figure 10:**
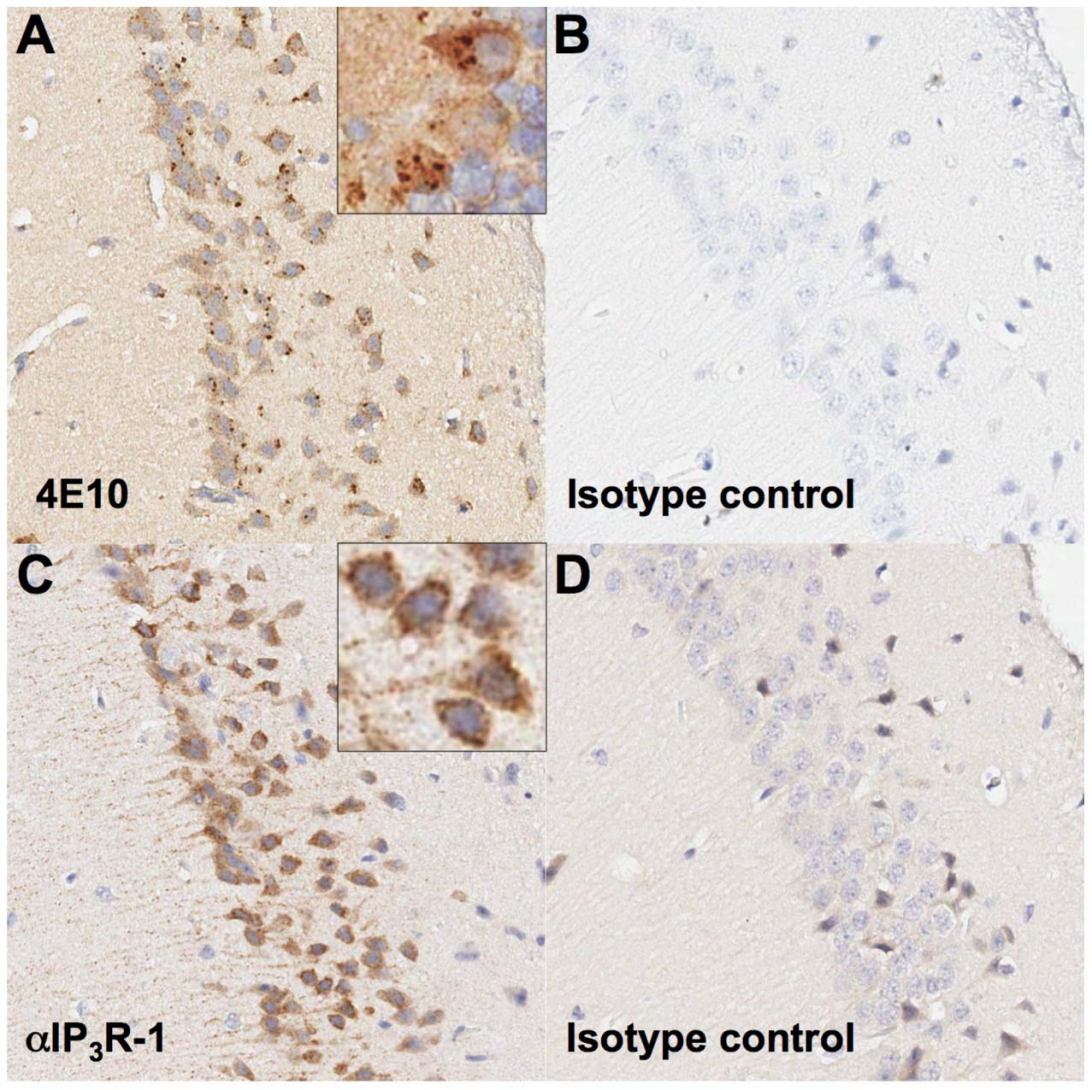
IHC of serial mouse cerebellar sections. (**A**) Immunohistochemical staining with 4E10 IgG in mouse cerebellum sections reveals perinuclear, nodular immunoreactivity in Purkinje neurons; inset shows a magnified view. (**B**) Human IgG Isotype control staining of mouse cerebellum is shown. (**C**) Staining for IP_3_R1 with an anti-IP_3_R1 IgG shows a perinuclear, nodular and diffuse cytoplasmic staining pattern in Purkinje neurons, mirroring that seen in (**A**); inset shows a magnified view. (**D**) Rabbit IgG isotype control staining of mouse cerebellum is shown.

Previous IHC studies of the three isoforms of IP_3_R [76] show that IP_3_R1 is markedly enriched in Purkinje neurons in the cerebellum, IP_3_R2 is overwhelmingly found in glial cells and IP_3_R3 is predominantly found in neurons, but not glia, and is enriched in neuropil, especially in neuronal terminals in limbic and basal forebrain regions. To further validate IP_3_R as the candidate 4E10 autoantigen, serial sections from fixed, paraffin-embedded mouse cerebellum were deparaffinized, rehydrated and treated to retrieve antigens, followed by staining with either 4E10 or an IHC-validated anti-IP_3_R1 rabbit polyclonal Ab (LifeSpan Biosciences LS-B2627) and appropriate secondary Abs. The commercial anti-IP_3_R1 polyclonal was raised against a synthetic peptide corresponding to C-terminal residues of human IP_3_R1, cross-reacts with mouse and rat orthologs and strongly stains Purkinje cells per the manufacturer's specifications (LifeSpan Biosciences). 4E10 strongly stained specific nodular substructures in a subset of neurons, identified as Purkinje cells on the basis of morphology, with minimal background staining of other cell types or substructures in these sections (**Fig. 10A and B**). The anti-IP_3_R1 polyclonal strongly stained a corresponding subset of neurons, also identified as Purkinje cells, and stained a combination of similar nodular structures as well as exhibiting more diffuse cytoplasmic and cellular process staining (**Fig. 10C and D**). SF3B3, together with splicing factor 3a and a 12S RNA, forms the U2 small nuclear ribonucleoprotein complex (U2 snRNP), which is localized to the nucleus [77]. The staining patterns of both 4E10 and the anti-IP_3_R1 polyclonal were inconsistent with recognition of antigens widely-expressed across cell types, like CL or SF3B3, or exclusively nuclear antigens, like SF3B3.

## Discussion

When applying “reverse vaccinology” [78] to the design of an HIV vaccine, it is important to determine whether or not the targeted bNAbs, *e.g.* 4E10, are autoreactive, and, if so, whether autoantigen recognition is separable from antigen recognition and neutralization. Despite usage of a breadth of murine light chains, 4E10H knock-in mice exhibited a profound blockade during B cell development consistent with the induction of central tolerance mechanisms, in which immature B cells expressing an autoreactive BCR are deleted (**Fig. 1**). In addition, the small residual population of B cells exported to the periphery, while able to respond to a variety of mitogenic signals, was unable to proliferate in response to IgM cross-linking. This phenotype substantiates the hypothesis that 4E10 is autoreactive. The questions then arise as to what autoantigen/s are mediating deletion and anergy and whether the mechanisms of 4E10 recognition of HIV and autoantigen epitopes are interdependent or separable, permitting the design of immunogens that generate Abs functionally equivalent to 4E10 with focused specificities that evade central and peripheral tolerance mechanisms.

**Figure 11:**
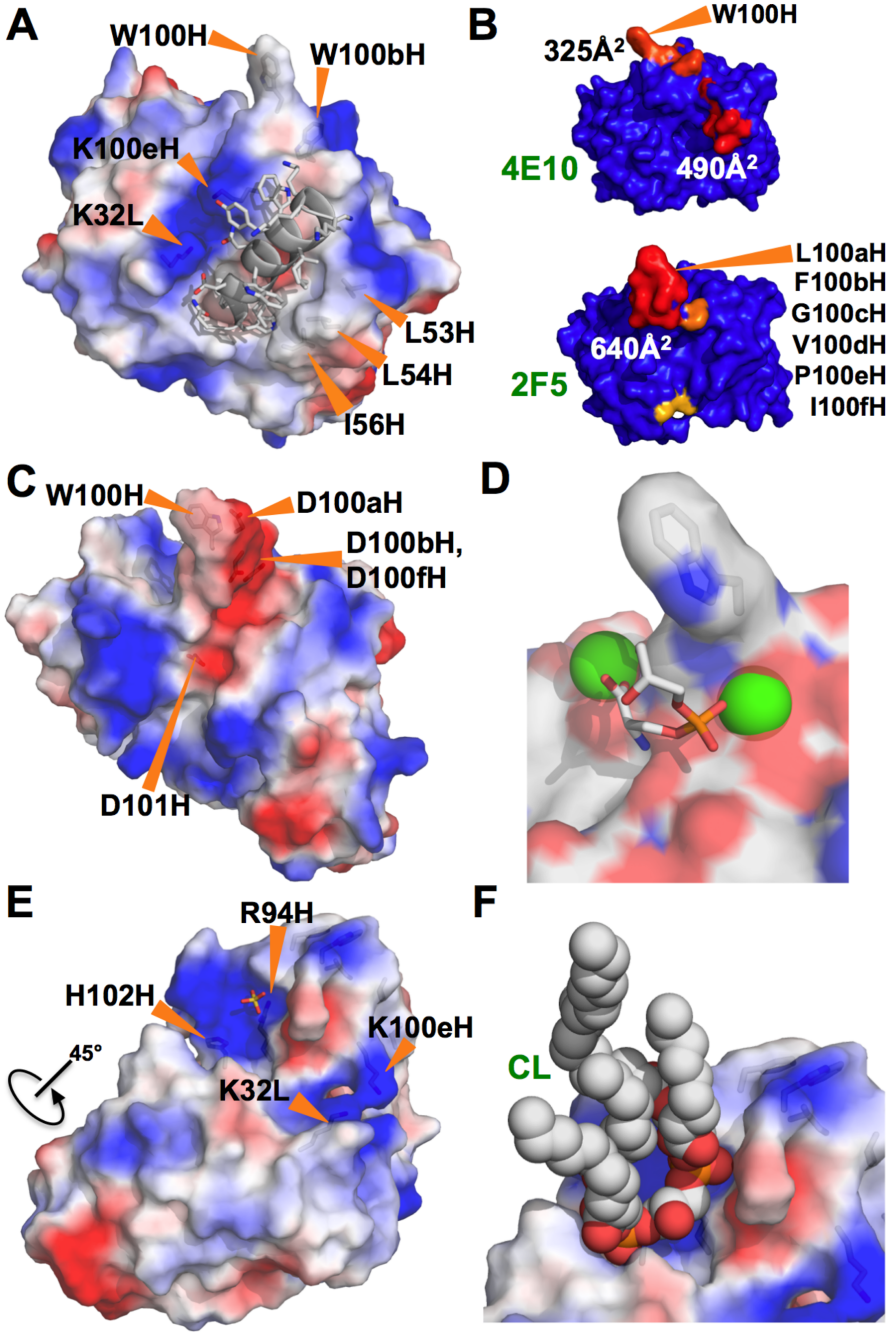
Structures of 4E10, b12, 2F5, the CL-binding yeast cytochrome bc1 complex and Annexin V. (**A**) The structure of the Fv domain of 4E10 (from 2FX7.pdb [9]) is shown in a semi-transparent molecular surface representation colored by electrostatic potential (blue: positive; red: negative). Side-chains of key residues are shown in licorice-stick representations and labeled. In this orientation, the V_L_ domain is in the upper left and the V_H_ domain is in the lower right. The backbone of the epitope peptide co-crystallized in this structure is shown in a cartoon representation, with side-chains shown in licorice-stick representations colored by atom-type (carbon: gray; oxygen: red; nitrogen: blue). (**B**) The molecular surfaces of the Fv domains of 4E10 (top) and 2F5 (bottom) are shown, oriented with V_L_ domains at left and the V_H_ domains at right. The surface is colored to show hydrophobic patches, defined by the program HotPatch [113]; patches are colored in descending order of total area (red, orange, yellow …). Key residues and hydrophobic patch areas are indicated. (**C**) The structure of the Fv domain of b12 (from 3RU8.pdb [104]) is shown as in (**A**). (**D**) Annexin V (1A8A.pdb [114]) is shown as a semi-transparent molecular surface colored by underlying atom type, highlighting the exposed tryptophan side-chain (shown in licorice-stick representation). Calcium ions, shown as green spheres, coordinate the phosphate moiety from PS (shown in a licorice-stick representation, colored by atom-type as in (**A**) plus phosphorus in orange). (**E**) The structure of the unbound form of 4E10 (4LLV.pdb) is shown as a semi-transparent molecular surface representation colored as in (**A**). Side-chains of key residues are shown in licorice-stick representations and labeled. The view of the molecule has been rotated roughly 45° from the orientation of 4E10 shown in (**A**). The coordinated SO_4_ ion is shown in a licorice-stick representation colored by atom type as in (**A**) plus sulfur in yellow. (**F**) CL is shown docked onto the unbound structure of 4E10 oriented and colored as in (**E**). Molecular images were generated with MacPyMOL [109].

Refined SPR analyses of Ab/liposome interactions (**Fig. 2**) confirmed that 4E10 binds to liposomes with compositions that include negatively-charged lipids (CL, PS), but not net neutrally-charged liposomes that contain only PC and cholesterol; by contrast, 2F5, b12 and ACLA show no binding to liposomes of any composition tested. The failure of ACLA to bind to CL-containing liposomes was likely accounted for by the absence, in these experiments, of required protein binding partners, like β2GPI, and draws a clear distinction between 4E10 binding behavior and autoimmune ACLA responses. The bivalent avidity of 4E10 for negatively-charged liposomes was weak compared to the univalent affinity of Annexin V binding. Crystal structures of 4E10/epitope peptide complexes [8]–[10], [34] limited how 4E10 might interact with lipids, liposomes or membranes (**Fig. 11A**). The MPER epitope peptide, structured as a short α-helix with a turn at the N-terminus, fits into a deep pocket in the Ab combining site, the back side of which consists of HCDR3. Two small hydrophobic patches are found centered on the side-chains of L53H, L54H and I56H, packing against a complementary hydrophobic face on the structured epitope peptide in complex structures, and W100H, which extends outwards from the tip of HCDR3. However, neither patch is particularly large or exceptional for Ab combining sites, as can be seen in comparison with 2F5 (**Fig. 11B**). Since 2F5 did not display liposome binding (**Fig. 2**), hydrophobic patches of this size order are not likely sufficient to account for 4E10 lipid binding.

The exposed side-chain of W100H on an extended HCDR3 structure, a feature which is shared with b12 (**Fig. 11C**), is positioned to potentially interact with membranes, but the side-chain of W100bH is partially buried behind the heavy chain N-terminus and the backbone of HCDR3 (**Fig. 11A**), rendering it inaccessible for docking to an encountered lipid bilayer. However, a sole exposed tryptophan side-chain on an extended HCDR3 is not sufficient on its own to confer liposome binding, otherwise b12 would also have displayed binding (**Fig. 2**), as it shares this feature with 4E10. We note that Annexin V also has a sole exposed tryptophan side-chain (**Fig. 11D**) which also does not support liposome binding in the absence of CL, PS or calcium (**Fig. 2**). Tryptophan side-chains can partition into lipidic phases, but typically influence the precise positioning of integral membrane proteins rather than confer membrane binding [79]–[81], consistent with our results. The sequence of the 4E10 HCDR3 segment is quite hydrophobic overall, but is mostly buried in the core of the protein (**Fig. 3A**). HCDR3 peptides from 2F5, b12 or 4E10, even when fully exposed as grafted Ubq-fusion constructs, did not measurably bind to liposomes of any composition tested, showing that hydrophobic, tryptophan-rich HCDR3 sequences are also insufficient to confer lipid binding out of context of an intact Ab.

Many examples of crystal structures showing specific CL recognition are available (*e. g.* cytochrome bc1 complexes, like 1P84.pdb [82]). In these structures, the acyl chains of CL are typically found wedged in hydrophobic grooves or packed against extensive hydrophobic faces, while the phosphoglycerol-phosphate head group of CL makes polar contacts with positively-charged residues on the protein, with CL bound in a deeply invaginated, highly shape-complementary site. However, SPR analyses of 4E10 binding to liposomes in response to increasing salt concentrations showed that the binding was dominated by electrostatic interactions (**Fig. 4A**), drawing a clear distinction with examples of specific CL recognition in protein crystal structures, which are dominated by apolar and van der Waals interactions within a subset of specific, polar bonds. The previously available 4E10 crystal structures, determined as complexes with epitope ligands, did reveal a potential phosphate binding site, between the side-chains of K32L and K100eH, albeit in the absence of a crevice or groove complementary to a full CL headgroup. However, mutational ablation of this site did not reduce binding to negatively-charged liposomes (**Fig. 4B**). No other candidate phosphate or CL binding sites were apparent from inspection of the complex crystal structures. Annexin V binds PS via a pair of metal coordination sites interacting with negatively-charged phosphate and carboxylate groups (**Fig. 11D**), hence membrane binding is calcium-dependent, but 4E10 liposome binding is not (**Fig. 2**), suggesting a different recognition mechanism; 4E10 is also not known to bind metal ions and does not possess recognizable metal binding sites.

The substantial reordering of the 4E10 combining site in the unbound state, however, uncovers an electropositive pocket large enough to accommodate various phospholipid headgroups, including the diphosphoglycerol headgroup of CL (**Fig. 11E**). An ordered sulfate ion occupies one end of this pocket in the crystal structure, designating a possible phosphate-binding subsite (SO_4_^2−^ and PO_4_^2−^ ions are chemically and structurally similar and can interact interchangeably within a protein binding site [83], [84]). CL coordinates were extracted from 1P84.pdb and modeled into this site by rigidly docking one of the CL phosphate moieties onto the position of the ordered sulfate ion and manually adjusting for optimal fit, which was easily achieved (**Fig. 11F**). This pocket readily accommodates the CL headgroup without clashes, with the second phosphate moiety making reasonable interactions with the side-chain of H102H, approximately 7 Å away from the sulfate site, and the main-chain nitrogens of Q1H and V2H. Docking CL in this manner orients the lipid tails outwards from the Ab, sterically accommodating docking to a negatively-charged liposome surface. While fitting CL well, the pocket does not display close shape complementarity, permitting other phospholipid headgroups to bind, consistent with the observed recognition degeneracy and weak absolute affinities. Because the residues involved in inferred CL contacts in this site all make important contributions to the overall structure of 4E10 in the complex structures, cleanly interpretable mutagenesis studies could not be performed.

We conclude that 4E10 is not specific for CL; rather, 4E10 weakly and non-specifically interacts with phospholipid headgroups or liposomes carrying a net negative charge primarily through electrostatic interactions associated with an electropositive pocket on the unbound conformer of 4E10. As displayed by the absence of binding to control liposomes, 4E10 does not show a general proclivity to indiscriminately bind liposomes, consistent with this conclusion. Such weak, non-specific, electrostatic binding of phospholipids is unlikely to be relevant to B cell tolerance induction nor is likely a requirement for neutralization, consistent with prior studies that showed reducing liposome affinity did not ablate neutralization [34], but may contribute generally to targeting of viral surfaces. However, since CL recognition is likely mediated by a pocket on the 4E10 combining site present only in the unbound form of 4E10, CL binding would directly compete with MPER epitope binding. In others words, 4E10 must release weakly bound CL before or concurrent with adopting the CDR conformation required to bind its MPER epitope and, therefore, cannot use membrane associations as a guide to direct epitope binding. The requirement to undergo a large conformational shift is consistent with the relatively slow association kinetics and weak overall affinity (*K*_D_ = 135 nM) observed when binding to Env proteins [34]; sampling an ensemble of CDR conformers may also contribute to the unusual biphasic association kinetics observed by SPR when binding liposomes.

Discarding CL from consideration, we utilized an unbiased proteomic approach to identify novel autoantigen candidates and to more fully characterize the polyreactivity of 4E10. A synthetic representation of the complete human proteome, composed of overlapping 36 amino acid peptides, was displayed on T7 phage and coupled with parallel DNA sequencing (PhIP-Seq) [45]. The PhIP-Seq method offers many improvements to prior methods, including the breadth of coverage and its quantitative nature [45]. 36-mers are long enough to retain substantial secondary and residual tertiary structure from the intact, folded, parent protein, potentially allowing identification of conformational epitopes. However, 36-mers are unlikely to represent stably folded species, and these peptides likely sample large conformer ensembles, including many non-native (non-self) states, increasing their utility for assaying polyspecificity. Since the peptides span a wide range of chemical properties (hydrophobic *vs.* hydrophilic, aromatic *vs.* charged *vs.* zwitterionic), the total conformer space also likely encompasses many peptide mimetics of non-protein species. A caveat with this approach is the difficulty in identifying discontinuous epitopes spanning more than one peptide, though linear segments of discontinuous epitopes may retain detectable binding. However, epitopes inaccessible in folded proteins, but which are exposed by proteolytic cleavage or other antigen processing events during B cell selection, can potentially be identified.

241 peptides were culled from the analysis because of uniform binding across a larger array of distinct Abs, but additional peptides retained in the analysis showed strong binding to more than one Ab in broader analyses not reported here, so binding results do not necessarily reflect just the specificity or polyspecificity of the Ab tested, but may also reflect the polyspecificity of a peptide. b12, an Ab that is presumably not autoreactive or unusually polyreactive, was analyzed to provide a baseline for PhIP-Seq results. The dozen top-scoring b12 peptides rising above the bulk response (**Fig. 8**) showed a common pattern of high positive charge and low sequence complexity, consistent with describing these peptides as poorly-ordered globs of positivity. The structure of b12 (**Fig. 11C**) is consistent with this observed, weak polyspecificity. Four aspartic acids adjacent to W100H, clustered on one face of the extended HCDR3 hairpin, form a large negatively-charged patch ideal for binding such peptides, at least weakly, as observed, and reveals a previously unrecognized aspect of b12 binding. The absence of extreme enrichments is consistent with the presumption that b12 is not self-reactive.

4E10, on the other hand, showed a strikingly different result (**Figs. 7B** and **8**), with highly-significant enrichment for 17 peptides well beyond the 10^−15^ *P*-value threshold set by b12, the top five with *P*-values ranging from 10^−58^ to 10^−255^, consistent with functional autoreactivity and limited, focused polyspecificity quite distinct from b12. There is no evidence that 4E10 displays an exceptional, low-avidity polyreactivity from these results, especially in comparison with b12. The 17 top-scoring 4E10 peptides, constituting novel candidate autoantigens, were moderately hydrophobic to hydrophobic in nature overall, with a range of net charge, consistent with ordered parts of folded proteins. The top-scoring 4E10 peptide is derived from IP_3_R2, with additional hits from two related IP_3_R types (IP_3_R1 and IP_3_R3) within the top-scoring five peptides (**Fig. 8**), providing evidence that one, two or all three IP_3_R types constitute candidate 4E10 autoantigens. None of the peptides from the top two scoring proteins from the ProtoArray 5 analysis [20] ranked in the top 232,000 peptides; the highest scoring peptide from the alternate proposed 4E10 autoantigen SF3B3 had a replicate-averaged −Log_10_ *P*-value of 1.54, ranking it at 143,927. We also note that of the top 17 4E10 PhIP-Seq peptides, only four, numbers 11 (WD repeat-containing protein 6), 12 (ras-GEF domain-containing family member 1A), 15 (UBX domain-containing protein 6 isoform 1) and 16 (complex I intermediate-associated protein 30, mitochondrial precursor), are from proteins represented on the ProtoArray 5, potentially accounting for the discordance in results between these two approaches. Scoring in PhIP-Seq also reflects internally-controlled peptide binding and not binding relative to a reference antibody, as in [20], which is necessarily affected by the binding profile of the reference antibody.

Three types of IP_3_R were identified as top-scoring PhIP-Seq hits and validated by SPR. In neuronal cell lines, IP_3_R1 and IP_3_R3 are expressed predominantly in the ER and perinuclear regions, while IP_3_R2 is expressed primarily in the nucleus [85]. IP_3_R3 shows an evenly diffuse cytoplasmic staining pattern, but the IP_3_R1 cytoplasmic pattern is more punctate, with strongly-staining nodular structures apparent [85]. However, failure to demonstrate binding of 4E10 to HEp-2 cells (which are not known to highly express IP_3_Rs) prevented comparative immunofluorescence analyses. We therefore sought to determine if the PhIP-Seq results identifying IP_3_R1, IP_3_R2 and IP_3_R3 as top candidate autoantigens were consistent with 4E10 recognition in a tissue-specific context by IHC. 4E10 staining of mouse cerebellar sections showed perinuclear staining of nodular structures specifically in Purkinje neurons, mirroring the staining of an anti-IP_3_R1 control polyclonal antibody. The 4E10 staining pattern observed was most consistent in terms of cell specificity with IP_3_R1 IHC patterns reported in previous studies [76], strongly arguing that IP_3_R1 is the lead candidate for a 4E10 autoantigen. The 4E10 staining pattern observed was also inconsistent with recognition of SF3B3. SF3B3, together with splicing factor 3a and a 12S RNA, forms the U2 small nuclear ribonucleoprotein complex (U2 snRNP) which is exclusively localized to the nucleus and widely-expressed across cell types [77].

Confirmation that 4E10 is autoreactive helps to identify the reasons vaccination strategies have failed, as tolerance mechanisms would limit the generation of anti-HIV Abs equivalent to 4E10. Our findings have important implications for future strategies to induce MPER-specific bNAbs. While lipid cross-reactivity mediated by HCDR3 had been suggested to play a role both in inducing B cell tolerance and in increasing neutralization potency through interactions with viral membranes, mutual functionalities precluding an engineered solution, our results alternately suggest that viral membrane binding is so weak and nonspecific, and competes with HIV epitope binding, that it is unlikely to be involved in the induction of tolerance mechanisms or to play a key role in neutralization. Weak but detectable binding of HCDR3 ubiquitin fusions to gp140s suggests that HCDR3 may make interactions to Env, rather than membrane, outside of the core 4E10 epitope. Neutralizing anti-influenza Abs (*e. g.* Abs CR6261, F10, FI6 and C179) often interact with a hydrophobic groove in the membrane proximal stalk region of hemagglutinin (HA) delineated by helix A and HA2 through hydrophobic HCDR residues [86]–[90]. The hydrophobic HCDR3 of 2F5, which recognizes an MPER motif neighboring the 4E10 epitope, has been argued to make similar contacts in the MPER region of Env as part of its neutralization mechanism [91]. We speculate that 4E10 may make analogous interactions to those seen in anti-HA Abs and 2F5, where 4E10 HCDR flexibility and hydrophobicity enable interactions with the presumed helical bundle of HIV gp41. However, the structure of the unbound form of 4E10 suggests that the long HCDR3 of 4E10 may also serve a role to shield hydrophobic patches in the combining site important for MPER recognition, prior to Env binding.

The fundamental concern in eliciting 4E10-like bNAbs was that viral epitope recognition would not be separable from autoantigen recognition. However, we showed that the viral epitope is not mimicking a self-antigen, because 4E10-selected sequences in the PhIP-Seq array analysis completely diverged from the 4E10 core epitope. The alternate core motif epitope from the likeliest candidate autoantigens, the three types of IP_3_R, was completely distinct from the 4E10 core epitope. Therefore, because the molecular mechanisms employed by 4E10 to recognize such divergent sequences are likely also distinct, even if they involve partially overlapping sites, it may be possible to ablate recognition of self-reactive epitopes while preserving interactions with neutralizing MPER epitopes. Future studies should concentrate on determining the complex crystal structure of 4E10 bound to the IP_3_R epitope in order to guide efforts towards engineering an immunogen that will elicit a non-autoreactive 4E10-like bNAb. However, if functional ontogeny of 4E10-equivalent responses requires engineering the ability to sample multiple, distinct conformers, in order to shield hydrophobic surfaces necessary for functional MPER recognition or to enable other molecular mechanisms necessary for recognition and/or neutralization, and if conventional Ab maturation typically proceeds by structural stabilization of the lone conformer optimal for epitope binding (*e. g.* [67]), it may not be possible to recapitulate 4E10-equivalent activities by any conventional immunization strategy.

## Materials and Methods

### Generation and analysis of 4E10H knock-in mice

4E10H targeted transgenic (knock-in) mice were generated by targeted insertion of a DNA construct encoding a mouse immunoglobulin heavy chain (IgH) promoter upstream of the rearranged Ab 4E10 IgH VDJ antigen-binding domain plus an mRNA splice donor sequence. The transgene was targeted to the mouse IgH genomic locus upstream of the immunoglobulin constant domains utilizing homologous recombination in C57BL/6 (B6) strain mouse embryonic stem (ES) cells. Transgene targeting was confirmed in ES cell clones by Southern blot (*data not shown*). 4E10H knock-in B6 ES cells were microinjected into FVB strain blastocysts, which were implanted into pseudo-pregnant female mice. Chimeric knock-in pups were identified by coat color and PCR genotyping, and crossed with B6 mice to generate pure B6 strain 4E10H knock-in mice. Germline transmission of the targeted 4E10H allele to progeny was confirmed by PCR genotyping (*data not shown*). Knock-in mice were inter-bred to establish homozygosity of the knock-in allele as assessed by PCR genotyping (*data not shown*). B6 background TgH(KL25) knock-in mice (referred to here as KL25H mice), expressing the mouse LCMV-neutralizing antibody KL25 rearranged IgH VDJ antigen-binding domain from the mouse IgH locus [46], were generously provided by Daniel Pinschewer (University of Geneva).

In order to analyze the composition of the bone marrow in transgenic animals, age-matched female wild type B6, KL25H knock-in, and 4E10H knock-in mice 6 to 8 weeks of age were euthanized and leg bones were isolated by dissection. Bone marrow was flushed from the leg bones with cold B cell media (RPMI/10% FBS/Penicillin/Streptomycin/2-mercaptoethanol) using a syringe and 18 g needle. Red blood cells were lysed with ACK lysing buffer (Gibco) and live cells were purified by density gradient centrifugation using Histopaque 1119 (Sigma-Aldrich). 5×10^6^ live cells from each mouse were stained with anti-mouse B220-Alexa647, IgM-PE, IgD-V450, and CD4-FITC (BD Biosciences), and analyzed on a FACS Canto II flow cytometer (BD Biosciences; **Fig. 1A**).

In order to analyze the ability of knock-in B cells to proliferate, splenocytes were purified from B6, KL25H, and 4E10H mice by density gradient centrifugation using Histopaque 1083 (Sigma-Aldrich). Purified cells were loaded with cell proliferation dye eFluor670 (eBioscience) according to the manufacturer's instructions. 1×10^6^ cells per well were incubated overnight in round-bottom 96-well plates with or without B cell stimuli (LPS, CpG DNA, an activatory anti-CD40 antibody, or anti-IgM F(ab′)_2_) in B cell media, in a 37°C CO_2_ cell culture incubator. The following day, cells were stained for cell surface markers (B220 and Thy1.2) and B cell proliferation was assessed by flow cytometry (**Fig. 1B**).

### Ethics statement

The activities involving the use of vertebrate animals described in herein have been reviewed and approved by the Fred Hutchinson Cancer Research Center (FHCRC) Institutional Animal Care and Use Committee (IACUC), file #1695. FHCRC has an approved Animal Welfare Assurance on file with the Office of Laboratory Animal Welfare (OLAW), Assurance number A3226-01.

### Liposome preparation

Lipids were purchased from Avanti Polar Lipids: bovine heart CL, 1,2-dioleoyl-sn-glycero-3-phosphoethanolamine (PE), egg L-α-phosphatidylcholine or 1-palmitoyl-2-oleoyl-sn-glycero-3-phosphocholine (PC), 1-stearoyl-2-oleoyl-sn-glycero-3-phosphoserine (PS), N-palmitoyl-D-erythro-sphingosylphosphorylcholine (SM), ovine wool cholesterol, 1,2-dioleoyl-sn-glycero-3-phosphoethanolamine-N-biotinyl (biotinylated PE). Control liposomes (4∶1 w/w PC∶cholesterol), CL liposomes (1∶7∶2 w/w CL∶PC∶cholesterol), and viral mimic liposomes (3.2∶5.4∶5.1∶4.8∶10.2 w/w cholesterol∶PC∶SM∶PE∶PS) [22], each containing 0.5% w/w biotinylated PE, were prepared by mixing each lipid component in chloroform, evaporating the solvent, and then resuspending at a final concentration of 1 mg/mL in phosphate buffered saline (PBS). In control and CL liposomes, PC was included as egg L-α-phosphatidylcholine, while in viral mimic liposomes, PC was included as 1-palmitoyl-2-oleoyl-sn-glycero-3-phosphocholine. Lipid solutions were incubated at 65°C for 5 min and then subjected to five freeze/thaw cycles alternating between a dry ice ethanol bath for 3 min and a 65°C water bath for 3 min with intermittent vortexing. Solutions were then passed through an extruder (Avanti) with a 0.1 µm polycarbonate membrane (Avanti) 21 times.

### Protein expression and purification

HCDR3-grafted Ubq fusion constructs (**Fig. 3**) with C-terminal His_6_-purification tags were engineered, cytoplasmically expressed in *E. coli* BL21(DE3) RIL cells (Invitrogen), and purified employing the methodology described in [92]. 4E10 [G(L50)E] IgG was produced as described in [34]. Seven of the top ten scoring 4E10 binding peptides identified in the PhIP-Seq analyses were cloned as fusion constructs with the human protein Scn [74] (**Fig. 9A**) using direct synthesis of human codon-optimized DNA (IDT) and Gibson cloning [93], [94] (GeneArt Seamless Cloning kit, Invitrogen) and expressed in HEK293 Freestyle cells (Invitrogen) using the Daedalus lentiviral transduction system [74]. Scn-peptide fusion constructs were purified by SEC from concentrated culture supernatants and validated by PAGE and Western analyses as described in [74] (*data not shown*).

### SPR interaction analyses

All SPR experiments were performed at 25°C on a Biacore T100 instrument with the T200 sensitivity enhancement (GE Healthcare). Liposome experiments (**Figs. 2**, **3D** and **4**) were run in 10 mM HEPES, pH 7.4, 150 mM NaCl and 0.5 mg/mL BSA (HBS-N-BSA) on SA sensor chips (GE Healthcare). Liposome preparations containing 0.5% biotinylated lipids were injected at 50 to 100 µg/mL, 5 µL/min, until approximately 1000 SPR response units (RUs) were captured, then allowed to equilibrate for four hours at 10 µL/min. Flow cell 1 was left blank as a reference surface. Single concentrations of 4E10 IgG (Polymun Scientific), 2F5 IgG (Polymun Scientific), b12 IgG (the kind gift of Pamela Bjorkman, Caltech), human ACLA polyclonal serum (LifeSpan BioSciences), and Annexin V (BD Biosciences) ±2.5 mM CaCl_2_, all at 300 nM (**Figs. 2** and **4**), or 4E10[G(L50)E] IgG [34] at 400 nM (**Fig. 4B**) and the three HCDR3-grafted Ubq-fusions at 1 µM (**Fig. 3D**), were injected in duplicate analyses at 50 µL/min for three minutes and allowed to dissociate for five minutes. In the salt series experiment (**Fig. 4A**), 4E10 IgG was diluted to 300 nM in HBS-N-BSA with added NaCl to 575, 320, or 150 mM total NaCl and analyzed in matched running buffers. HIV gp140 SF162 trimers [34] at 30 µg/mL in 10 mM sodium acetate (pH = 5.0) were immobilized at a density of 3250 RUs on a CM5 sensor chip (GE Healthcare) using standard amine coupling chemistry (**Fig. 3C**). An activated/deactivated flow cell was used as a reference surface. HCDR3-grafted Ubq-fusions were injected in duplicate experiments in 10 mM HEPES, pH 7.4, 150 mM NaCl, 3 mM EDTA, 0.05% (v/v) surfactant P20 (HBS-EP+) at 1 µM and 5 or 10 µM (depending on maximum responses) at 50 µL/min for three minutes and allowed to dissociate for six minutes (**Fig. 3C**). Approximately 3000 RUs of each Scn-peptide fusion construct or a Strep-tagged Scn control [74] were immobilized on CM5 chips (GE Healthcare) using standard amine coupling chemistry and HBS-EP+ as running buffer (**Fig. 9**); optimal immobilization conditions were determined for each fusion by pH scouting. DTT was included in the immobilization buffer for fusions 1 and 3 to prevent oxidation of cysteines in the 36-mer peptide sequences. Duplicate 4E10 IgG samples at 300 nM were injected at 50 µL/min for three minutes and allowed to dissociate for 15 minutes in HBS-EP+ or HBS-EP+ at 500 mM NaCl. In order to determine the specific activity of the fusion-coupled surfaces, each Scn-peptide fusion surface was saturated with StrepMAB-Immo IgG (IBA BioTAGnology) by making three five minute injections of 100 nM IgG at 30 µL/min. Final SPR responses of 4E10 IgG binding to Scn-peptide fusions (**Fig. 9**) were normalized in Scrubber 2.0b (Biologic) software by comparing the observed specific activities (fusion #1: 13%; fusion #2: 14%; fusion #3: 29%; fusion #4: 12%; fusion #5: 34%; Scn controls: 51% or 40%). Specific activities are reduced from 100% by either steric occlusion through coupling or proteolytic degradation of the Scn-peptide fusions. All SPR data were double-referenced [49] using BiaEval version 2.0.3 (GE Healthcare) and plotted with Prism 5.0d for Mac OSX software.

### Crystallography

Crystals of ligand-free 4E10 Fv [34], expressed without purification tags using the Daedalus system [74], were grown at 25°C by the hanging-drop vapor-diffusion method with a well solution of 0.8 M Li_2_SO_4_ buffered with 0.1 M NaOAc (pH = 4.6). Crystals grew within one week and were cryopreserved in a mother liquor of well solution plus 15% v/v glycerol. Diffraction data were collected with both CuKα radiation on an in-house R-AXIS IV++ image plate detector with HR optics (Rigaku) at −170°C and at the Advanced Light Source beamline 5.0.2 (ALS; Lawrence Berkeley National Laboratory, Berkeley, CA) and reduced using HKL-2000 [95]. Initial phase information was determined by molecular replacement with Phaser [96], as implemented in the CCP4i program suite [97], a previous bound 4E10 structure (3H3P.pdb [34]), with CDRs deleted, as a search model. Phases were improved by subsequent rounds of model building and refinement using COOT [98] and REFMAC [99] with the ALS data. Eight TLS groups, one for each heavy and light chain, were used during refinement [100], [101]. Structure validation was carried out with the MolProbity server [102], and the RCSB ADIT validation server. An anomalous difference Fourier synthesis, using in-house native data reduced without merging Bijvoet pairs, was calculated to verify SO_4_ placement and the initial correctness of the molecular replacement solution. Crystallographic statistics are reported in **Table 1** and the final coordinates have been deposited in the PDB [103], accession code 4LLV.

### PhIP-Seq analysis

For each of the two Abs analyzed, 3 mg of magnetic beads (Invitrogen Dynabeads M-270 Epoxy) were resuspended in 60 µL 0.1 M NaPO_4_, pH 7.4. Beads were rocked at ambient for 24 hrs with 60 µg of either 4E10 or b12 IgG in 1 M (NH_4_)_2_SO_4_ and then washed with 10 mM glycine in PBS to cap unreacted epoxy groups. Activity of 4E10 and b12 beads was confirmed by epitope-scaffold [92], [104] binding prior to PhIP-Seq analyses. PhIP-Seq analyses were performed as described in [45], [105]. Briefly, 4E10 and b12 beads were incubated with the T7-Pep library. DNA from immunoprecipitated phage was recovered and sequenced. Two replicate experiments were performed for both 4E10 and b12 and the enrichment significance for each clone was calculated as −Log_10_ *P*-values.

### IHC

Four micron sections of formalin-fixed and paraffin-embedded tissues were cut and baked for 1 hr at 60°C, deparaffinized with xylene and rehydrated in a series of graded ethanol solutions (100%, 95%, and 80%) followed by distilled water. Antigen retrieval in pH 6.0 Citrate Buffer (Dako Target Retrieval Solution S1699) was then performed for 5 min in a pressure cooker. All of the following incubations were performed at room temperature on a Dako Autostainer Plus. Wash buffer (0.05M Tris, 0.3M NaCl, and 0.1% Tween-20, pH 7.2–7.6) was used between all subsequent steps except between the protein block step and application of the primary Ab. A 3% v/v hydrogen peroxide block step was done for 8 min. IP_3_R1 staining: a protein block step consisting of 0.05M Tris, 0.15M NaCl, 0.25% Casein, 0.1% Tween 20, pH 7.6 (TCT Buffer) was performed for 10 minutes. Tissues were then stained with polyclonal rabbit anti-human IP_3_R1 IgG (LifeSpan Biosciences) at a dilution of 1∶200 (2.5 µg/mL) or rabbit IgG matched to the protein concentration of the primary Ab for 60 minutes. Mach 2 Rabbit HRP-polymer (Biocare Medical RHRP520L) was applied for 30 minutes. 4E10 staining: avidin block (Biocare Medical AB972M-A) was applied for 10 minutes, followed by biotin block (Biocare Medical AB972M-B) for 10 minutes. Next, a protein block step (TCT Buffer plus 15% goat serum) was performed for 10 minutes. Tissues were then stained with 4E10 IgG (Polymun AB004) at a dilution of 1∶100 (10 µg/mL) or normal human serum matched to the protein concentration of the primary Ab for 60 minutes. The Fab of a biotinylated goat anti-human secondary Ab (Jackson Laboratory 109-067-993) was applied for 30 minutes followed by incubation with streptavidin-HRP (Jackson Laboratory 016-030-084) at a dilution of 1∶2000 for 30 minutes. Finally, a DAB+ substrate-chromogen incubation was done (Dako K3468) for two applications of 4 min each. The slides were counterstained with Automation Hematoxylin (Dako S3301) for 2 minutes and then coverslipped.
